# Antimycobacterial Activity and Safety Profile Assessment of* Alpinia galanga* and* Tinospora cordifolia*

**DOI:** 10.1155/2018/2934583

**Published:** 2018-07-08

**Authors:** Mohamed F. Alajmi, Ramzi A. Mothana, Adnan J. Al-Rehaily, Jamal M. Khaled

**Affiliations:** ^1^Department of Pharmacognosy, College of Pharmacy, King Saud University, P.O. Box 2457, Riyadh 11451, Saudi Arabia; ^2^Departments of Botany and Microbiology, College of Science, King Saud University, Riyadh 11451, Saudi Arabia

## Abstract

Tuberculosis (TB) remains a common deadly infectious disease and worldwide a major health problem. The current study was therefore designed to investigate the* in vitro* antimycobacterial activity of different extracts of* Alpinia galanga* and* Tinospora cordifolia*. Moreover, a safety assessment for both plants was carried out. Dichloromethane and ethanolic extracts of each plant were examined against H37Rv INH-sensitive and resistant INH strains of* Mycobacterium tuberculosis*. The safety assessment of both plants has been performed through* in vivo* acute and chronic toxicity studies in animal model. Body weight, food consumption, water intake, organ's weight, and haematological and biochemical parameters of blood and serum were evaluated. The extracts of* A. galanga* and* T. cordifolia* produced significant and dose-dependent inhibitory activity with maximum effect of 18-32% at 50 *μ*g/ml against both strains of* M. tuberculosis*. No effect on the body weight or food and water consumption was observed but* A. galanga* caused significantly an increase in the relative weight of the heart, liver, spleen, and kidney. Haematological studies of both plants revealed a slight but significant fall in the RBC and WBC level as well as haemoglobin and platelets. In addition,* A. galanga* extracts increased significantly liver enzymes and bilirubin and glucose.

## 1. Introduction

Tuberculosis (TB) is one of the global health problems and leading causes of human morbidity and mortality [1, 2]. TB is recognized as one of the top 10 causes of death worldwide. The World Health Organization (WHO) estimated 10.4 million people fell ill with TB in 2016 [3]. In 2016, there were an estimated 1.3 million TB deaths among HIV-negative people and an additional 374 000 deaths among HIV-positive people. Over 95% of TB deaths occur in low- and middle-income countries [3]. Although the current treatment of TB with drug combination of isoniazid, rifampicin, ethambutol, and pyrazinamide has a high success rate, drug-resistant TB is a continuing threat [4, 5]. In 2016, there were 600 000 new cases with resistance to rifampicin (RRTB), the most effective first-line drug, of which 490 000 had multidrug-resistant TB (MDR-TB) [3]. Consequently, the need to develop new novel tuberculosis drugs which are less toxic and more effective against resistant* Mycobacterium* strains is imperative. Natural products, either as pure isolated compounds or as extracts, represent a promising source of novel drugs for the treatment of various diseases including TB [5, 6]. Therefore, we were encouraged to investigate two well-known medicinal plants, namely,* Alpinia galanga *and* Tinospora cordifolia*.

*Alpinia galanga* (L.) Willd. (Zingiberaceae) known as Galangal, a member of the ginger family and native to Southern China and Thailand, is primarily used as a flavoring especially in the preparation of fresh Thai curry paste and Thai soup [7]. It is widely cultivated in Southeast Asian countries, e.g., China, Indonesia, Thailand, India, and Philippines [8]. Galangal exhibited different pharmacological activities such as antimicrobial, anti-inflammatory, carminative, antipyretic, aphrodisiac, and emmenagogue and traditionally has been used for the treatment of various diseases such as kidney disorders, diabetes, cough, tuberculosis, bronchitis, rheumatism, asthma, and heart diseases [8–12].

*Tinospora cordifolia *(Willd.) Miers which belongs to the family Menispermaceae is a climbing shrub widely distributed in tropical areas in South East Asia, e.g., India, China, Myanmar, Sri Lanka, Indonesia, Malaysia, Thailand, and Philippines [13]. It is introduced in Saudi Arabia from Pakistan and grown as ornamental. In Indian colloquial, this plant is known as Giloya, meaning elixir of paradise, which kept celestial beings young and saved from aging [14]. The plant has significant medicinal importance and is widely used in the Indian folk medicine for increasing the lifespan, promoting intelligence, and improving memory and as antiaging agent [13–15]. Recent studies on* T. cordifolia* reported antioxidant, radical scavenging, hepatoprotective, anticancer, antiallergic, immunmodulatory, and anti-inflammatory effects [16–21]. The current study was therefore designed to investigate the antimycobacterial activity of different extracts of* Alpinia galanga* and* Tinospora cordifolia*. Moreover, a safety profile assessment of both plants has been carried out through* in vivo* acute and chronic toxicity studies in animal model.

## 2. Materials and Methods

### 2.1. Plant Materials

The plant materials, namely,* Alpinia galanga *(roots and rhizomes) and* Tinospora cordifolia* (leaves and stems) were purchased from India, Dawa Khana Tibbiya College, Aligarh Muslim University, Aligarh. The plants were authenticated by Professor S.H. Afaq, Department of Ilmul Advia, Ajmal Khan Tibbiya College, Aligarh Muslim University, Aligarh. A voucher specimen for each plant was preserved in the lab for further documentation.

### 2.2. Extraction of the Plants

The air-dried and powdered plant materials were defatted with petroleum ether. Then the plant materials were percolated separately with dichloromethane in 10 L percolator for one day. The process was repeated till the exhaustion of the plant materials. Then the obtained extracts were collected, combined, filtered, and evaporated using a rotary evaporators (Buchi® evaporator, Switzerland) at 40°C under vacuum. Then, the same extraction process was done for both plant materials using ethanol 96%. The extracts were then preserved at 4°C until testing.

### 2.3. Phytochemical Screening

Thin layer chromatography was developed for each plant's extract using different mixtures of organic solvents as mobile phases, visualized under UV (254 and 366 nm), and sprayed with various chemical reagents, e.g., anisaldehyde-sulfuric acid for terpenoids, Dragendorff's reagent for alkaloids, Borntrager reagent for anthraquinones, etc. according to previously published methodology [22].

### 2.4. Determination of the Antimycobacterial Activity

BD Bactec ™ MGIT 960 kit (Becton, Dickinson and Company, USA) was used for antimycobacterial susceptibility testing of* Mycobacterium tuberculosis.* The Bactec MGIT 960 kit was used with Bactec MGIT System.

#### 2.4.1. Mycobacterial Strains/Isolates

The* in vitro* antimycobacterial activity of the extracts was carried out against two* Mycobacterium tuberculosis* strains, that are H37Rv INH-sensitive and resistant INH strains. Samples were prepared at 5 mg/ml in DMSO by sonicating and vortexing as needed and then added to Middlebrook 7H12 media and serially diluted 2-fold within 96-well plates in a volume of 100 *μ*l per well.* M. tuberculosis* H37Rv was then added in a volume of 100 *μ*l 7H12 to achieve a bacterial density of approximately 1 x 10^5^ CFU/ml. The highest final concentration of the samples was therefore 50 *μ*g/ml with a maximum final DMSO concentration of 1% v/v.

### 2.5. Acute Toxicity Study

For acute studies, Swiss albino mice (home bred) aged 6–7 weeks, weighing about 24–28 g, were taken from Animal Care Centre (College of Pharmacy, King Saud University) and fed on Purina Chow diet and water ad labium, were used in this study. The animals were maintained under controlled temperature, humidity, and automated light cycles (12 h light, 12 h dark). The protocol of the current study (CBR 4537) was approved by the Ethics Committee of the Experimental Animal Care Society, College of Pharmacy, King Saud University, Riyadh, Saudi Arabia. According to the test guideline of Organization for Economic Cooperation and Development (OECD), the toxicity tests were carried out [23].

### 2.6. Toxicity Study Design

For the determination of the acute toxicity, mice (males) were randomly divided into different groups (N=6-10). Different doses of each test extract (0.5, 1, 2, 5, 8, and 10 g/kg) were administered intraperitoneally. The extracts were suspended in 0.2% aqueous Tween 80 or 0.25% carboxymethyl cellulose. The animals were observed for 72 h for signs of toxicity and mortality and LD_50_ was calculated according to published method [24].

### 2.7. Chronic Toxicity Study

A total of 40 male and 40 female Swiss albino mice were randomly allocated to the control and test groups. The extract in each case was mixed with drinking water for feasibility of administration due to long treatment duration. The dose selected was 1/40^th^ of the LD_50_. The treatment was continued for a period of 12 weeks [24]. The food consumption and water intake recorded weekly. The body weights of animals recorded shortly before the administration of the tested extracts and at the end of each week. The animals were then observed for all external general symptoms of toxicity, body weight changes, and mortality. The average pre- and posttreatment body weights, vital organ weights of the treated animals, were compared with the control group. Ten male and ten female rats were used in each group having one control and two treated groups. One group of treated female rats were mated with treated males and pregnancy outcomes were studied. Urine was collected 1-2 days before the end of the treatment. The treated animals were fasted for 12 h and then anesthetized. Blood samples were collected via heart puncture and centrifuged at 3000 rpm for 10 min. The plasma was then stored at -20°C pending for analysis of the biochemical parameters. Vital organs were removed, weighed, and investigated for apparent signs of toxicity and stored in 10% formalin for histological studies. The percentage of each organ relative to the body weight of the animal was calculated.

### 2.8. Haematological Studies

Whole noncentrifuged blood was used for determination of some haematological values. The blood was analyzed for WBC and RBC count, haemoglobin, platelets, neutrophils, and lymphocytes measurement using Contraves Digicell 3100H (Zurich).

### 2.9. Serum Analysis of Biochemical Parameters

A colorimetric method was used for the determination of the biochemical parameters (AST, ALT, GGT, ALP, bilirubin, glucose, lipid profile, and total protein) in plasma. The enzyme activity was quantified spectrophotometrically using commercial enzymatic kits (Crescent Diagnostics Test Kits, SA) [25].

### 2.10. Statistical Analysis

The results were presented as mean ± standard error of the mean (SEM). Statistical differences were analyzed using ANOVA with Dunnett as posttest. A value of p < 0.05 was considered statistically significant [26].

## 3. Results

As presented in Table 1, the yield of the dichloromethane extracts is more than the ethanolic extracts of both plant species. The results of the phytochemical screening in Table 1 indicated different types of active constituents in* Alpinia galanga* such as essential oil with terpenoids and flavonoids, while* Tinospora cordifolia *indicated the presence of terpenoids, alkaloids, and flavonoids.

### 3.1. Antimycobacterial Activity

**Figure 1:**
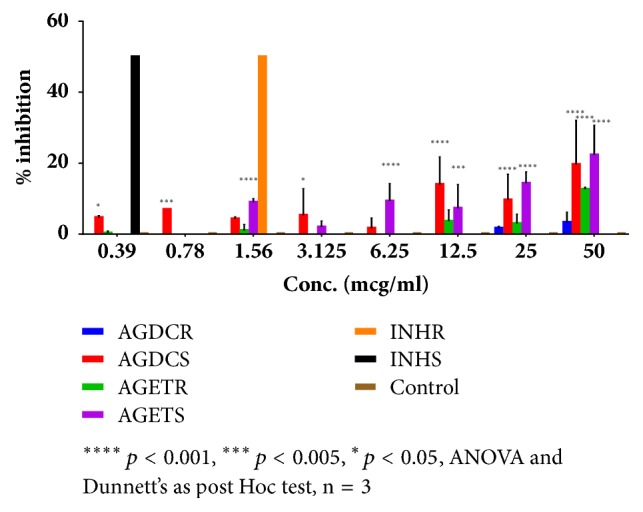
Effect of* Alpinia galanga* dichloromethane extract (AGDC) and* Alpinia galanga* ethanol extract (AGET) on sensitive strain (AGDCS and AGETS) and resistant strain (AGDCR and AGETR) of* Mycobacterium tuberculosis *(MT). Results presented as mean % inhibition ± SD and compared to control nontreated MT.

As depicted in Figure 1, whereas ethanolic and dichloromethane extracts of* Alpinia galanga* (AGET and AGDC) produced significant (*p *< 0.001) and dose-dependent inhibitory activity against sensitive strains of* Mycobacterium tuberculosis* (MT), the negative control (DMSO) did not show any effect on the bacterial growth. AGET exhibited a significant (*p *< 0.001) and dose-dependent inhibition on sensitive strains of MT. Maximum inhibitory effect was shown at 50 *μ*g/ml (22.3%, Figure 1). Moreover, AGET produced a significant (*p *< 0.001) inhibitory effect on resistant strains of MT only at the highest concentration 50 *μ*g/ml (12.7%, Figure 1). In addition to that, Figure 1 demonstrated that AGDC showed significant (*p *< 0.001) dose-dependent inhibitory effect only on sensitive strains of MT with maximum effect at of 19.7% at 50 *μ*g/ml.

**Figure 2:**
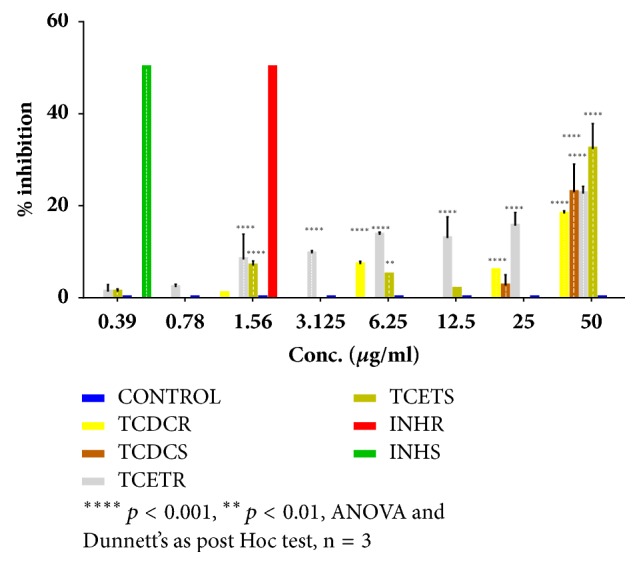
Effect of* Tinospora cordifolia* dichloromethane extract (TCDC) and* Tinospora cordifolia* ethanol extract (TCET) on sensitive strain (TCDCS and TCETS) and resistant strain (TCDCR and TCETR) of* Mycobacterium tuberculosis *(MT). Results are presented as mean of per cent inhibition ± SD and compared to control nontreated MT.

The results of the inhibitory effect of* Tinospora cordifolia* ethanolic and dichloromethane extracts (TCET and TCDC) against sensitive and resistant MT are shown in Figure 2. Figure 2 demonstrated that TCET produced a significant (*p* < 0.001) and dose-dependent inhibitory effect against both sensitive and resistant strains of MT with maximum effect of 32.3% and 22.7% at 50 *μ*g/ml, respectively. Furthermore, TCDC showed a significant (*p* < 0.001) antimycobacterial effect against the sensitive strain of MT with maximum effect of 23% at 50 *μ*g/ml (Figure 2). It also produced significant (*p* < 0.001) and dose-dependent inhibitory effect against the resistant strain of MT with maximum effect of 18.3% at 50 *μ*g/ml (Figure 2).

### 3.2. Effect of the Extracts in Acute Toxicity Test.

The administration of a dose lower than 5 g/kg of all extracts did not show any mortality or observable symptoms. However, gross behavioural changes such as increased heart rate, convulsion, twitches, itching and excitation, mortality, and other signs of toxicity manifestations were observable with the highest doses (5, 8 and 10 g/kg). The treatment of the animals with doses ≥ 5 g/kg of* A. galanga *dichloromethane extract (AGDC) produced increased respiration, pilo erection, Straub tail, tremors, increased muscle tone, and sedation, while* A. galanga *ethanolic extract (AGET) produced defecation, writhing, sedation, and calmness. The administration of* T. cordifolia *ethanolic extract (TCET) increased heart rate and CNS excitation (convulsion, twitches, and itching). In addition, both extracts of* T. cordifolia *produced defecation and writhing. The mortality rate and LD_50_ values are demonstrated in Table 2. As depicted in Table 2, the most toxic extract was AGDC (6.6 g/kg) followed by the TCET (6.83 g/kg). The rest of the extracts were relatively safer with LD_50_ values of 7.5–7.7 g/kg (Table 2).

### 3.3. Effect of the Extracts on Body Weight, Food, and Water Consumption and Organ's Weight in Chronic Oral Toxicity Test

During the treatment period of 12 weeks, the body weight has increased gradually in the control and extracts treated female and male mice groups (data are not shown). The percentage of increase in body weight of the* A. galanga* and* T. cordifolia* extracts treated mice was not significantly different compared to the control mice. In addition, the food and water consumption of the* A. galanga* and* T. cordifolia* extracts treated female and male mice exhibited no significant difference compared to the control mice (data are not shown). On the other hand,* A. galanga* extracts (AGDC and AGET) increased significantly (*P* < 0.05) the relative weight of the heart, liver, lungs, spleen, kidney, and testis. No significant changes in organ's weight were noted for the* T. cordifolia* extract TCDC. However, the* T. cordifolia *extract TCET increased significantly (P < 0.05) the weight of liver and kidney (Table 3).

### 3.4. Effect of the Extracts on Haematological Parameters in Chronic Oral Toxicity Test

The haematological analysis from control and treated animal groups for the chronic toxicity study is shown in Table 4. Treatment with* A. galanga* extracts AGDC at 166 mg/kg and AGET at 193 mg/kg showed variable changes in the haematological parameters, while AGDC and AGET decreased significantly (*P* < 0.01) red and white blood cells count and only AGET decreased significantly (*P* < 0.01) haemoglobin and number of platelets. Treatment with* T. cordifolia* extract TCET at 170 mg/kg showed a significant decrease (*P* < 0.01) in red and white blood cells count, haemoglobin, and lymphocytes (Table 4), whereas TCDC only deceased number of platelets significantly (*P* < 0.05).

### 3.5. Effect of the Extracts on Biochemical Parameters in Chronic Oral Toxicity Test

For the biochemical parameters, the administration of* A. galanga* extract AGDC at 166 increased significantly AST (*P* < 0.05), ALT, GGT, ALU, bilirubin, and blood glucose level (*P* < 0.001) (Table 5). In addition, the treatment with AGET at 193 mg/kg showed a significant increase of ALT, ALT and bilirubin (*P* < 0.01), GGT (*P* < 0.01), and ALU (*P* < 0.05). Furthermore, as demonstrated in Table 5, the administration of* T. cordifolia* extract TCDC caused a significant decrease of GGT (*P* < 0.001) and blood glucose level (*P* < 0.01), whereas TCET showed a significant increase of AST and bilirubin (*P* < 0.01) and ALU (*P* < 0.05) (Table 5). In addition to that, both plants exhibited a significant increase (*P* < 0.01) of sodium, potassium, and creatinine level (Table 5).

### 3.6. Effect of the Extracts on Lipid Profile and Total Protein in Chronic Oral Toxicity Test

As shown in Table 6, the treatment with* A. galanga* extract AGDC at 166 increased significantly (*P* < 0.001) the level of cholesterol, triglycerides, HDL, and VLDL and decreased significantly (*P* < 0.001) the total protein compared to the normal control group. On the contrary, the administration of AGET significantly (*P* < 0.05) decreased the level of lipid profile compared to the normal control group (Table 6). In addition, the administration of TCDC significantly (*P* < 0.01) reduced the level of all lipid profile compared to the normal control group, whereas TCET caused a significant (*P* < 0.01) elevation of cholesterol, triglycerides, HDL, and VLDL and a significant (*P* < 0.05) reduction of LDL and total protein (Table 6).

## 4. Discussion

Tuberculosis (TB) remains a common deadly infectious disease and worldwide a major health problem. The World Health Organization (WHO) estimated 10.4 million people fell ill with TB in 2016 [3]. In 2016, WHO reported about 1.5 million TB deaths. TB ranks as the second leading cause of deaths among infectious diseases after HIV [27]. The current combination therapy with antimycobacterial drugs like isoniazid, rifampicin, streptomycin, and ethambutol causes various side effects mainly hepatotoxicity. Moreover, the development of multidrug-resistant bacterial strains made the treatment more difficult and complicated. Consequently, there is an urgent need for novel, more effective, with lower side effects, and less expensive drugs. Keeping these aforementioned facts in mind, medicinal plants have received more attention as a potential source in drug discovery against TB [28]. In the current study, we examined the* in vitro* antimycobacterial activity of four extracts obtained from two medicinal plants namely* Alpinia galanga* and* Tinospora cordifolia* against two* Mycobacterium tuberculosis* strains, that are H37Rv INH-sensitive and resistant INH strains. The present work was further extended to evaluate the safety profile of both* A. galanga* and* T. cordifolia* whereas* in vivo* assessment of the acute and chronic toxicity in animal model has been performed. Criteria for selection these plant species for investigation are the traditional uses to treat cough and other respiratory tract conditions including tuberculosis [8, 11, 13, 15] as well as the previously reported antimicrobial activity against various bacterial strains [8, 9, 15, 29, 30].

In general the H37Rv INH-sensitive* M. tuberculosis* strain showed more susceptibility than the resistant INH strain to the investigated extracts. Our obtained results revealed that the ethanolic and dichloromethane extracts of* A. galanga* (AGET and AGDC) possessed a considerable significant antimycobacterial activity at the highest concentration tested (50 *μ*g/ml). Our data are in agreement with previously published reports on* A. galanga* [8, 31]. In the study carried out by Soundhari and Rajarajan [31], it was shown that* A. galanga* has an antimycobacterial activity against isoniazid-resistant strain with MIC-value of 250 *μ*g/ml, whereas Gupta and coworkers [8] described a bactericidal activity against* M. tuberculosis* under axenic aerobic conditions at 50-100 *μ*g/ml. The variation in the active concentrations may be attributed to differences in the methods used in extraction and the assay used for the evaluation of the antimycobacterial activity. A recent published study done by Warit and coworkers [32] reported the evaluation of the antituberculosis activity of one of the major compounds in* A. galanga*, namely, 1'-acetoxychavicol acetate and its enantiomers. It was shown that the* S*-enantiomer of 1'-acetoxychavicol acetate has a remarkable antimycobacterial activity against H37R*a* and H37R*v* strains with MIC values of 0.2 and 0.7 *μ*g/ml, respectively. There is little data in the literature about antimycobacterial activity of* Tinospora cordifolia*. To the best of our knowledge this is the first report on the antimycobacterial activity of* T. cordifolia* against H37Rv INH-sensitive and resistant INH strains. The results of the inhibitory effect of* T. cordifolia* ethanolic and dichloromethane extracts (TCET and TCDC) against sensitive and resistant* M. tuberculosis *revealed a considerable significant antimycobacterial activity at the highest concentration tested (50 *μ*g/ml). Our obtained result was indirectly in agreement with a recently published study by Gupta and coworkers [33] who reported the isolation of a polysaccharide from* T. cordifolia* which inhibited the survival of* M. tuberculosis* by controlling influence on host immune responses. It is assumed that this modulation would improve the therapeutic efficacy of currently used antituberculosis drugs and offer an interesting strategy for the development of other choice of treatments to control this disease.

Our phytochemical screening showed the presence of terpenoids, essential oils, phenolic compounds (phenylpropanoids), and flavonoids in* A. galanga*. These results are in agreement with data reported on the chemistry of* A. galanga* [34–37]. Moreover, our data revealed the presence of terpenoids, alkaloids, and flavonoids in* T. cordifolia*. These results are also in agreement with reports on preliminary phytochemical screenings of this plant [19, 38, 39]. We assume that the displayed antimycobacterial activity could be attributed to such classes of natural compounds which may contribute together in growth inhibition of* M. tuberculosis*.

Flavonoids isolated from* Erythrina schliebenii* were studied for antimycobacterial activity against* M. *tuberculosis (H37Rv strain) and exhibited MIC values 36.9-101.8 *μ*M [40]. A recent study demonstrated that isorhamnetin possessed antimycobacterial activity against multidrug- and extensively drug-resistant clinical isolates of H37Rv strain of* M. *tuberculosis, with MIC values of 158 and 316 *μ*M, respectively [41]. In addition, 3-cinnamoyltribuloside, tribuloside, afzelin, and astilbin which were isolated from* Heritiera littoralis* showed antimycobacterial activity against* Mycobacterium madagascariense* and* Mycobacterium indicus pranii*, with MIC values in the range of 0.8-1.6 mg/ml [42, 43].

Furthermore, terpenoids, which were isolated from various natural sources, e.g., medicinal plants, fungi, and marine organisms also displayed a promising and interesting antitubercular activity against different strains of* M. tuberculosis* [43–47]. Recently, Isaka and coworkers [44, 45] reported a potent antitubercular activity of several lanostane triterpenoids isolated from different cultures of* Ganoderma* species with MIC values ranging between 0.78 and 12.5 *μ*g/ml. In addition, a lot of isolated alkaloids belonging to various classes, e.g., indole, pyrrole, indoloquinoline, carbazole, manzamine, quinoline, isoquinoline, and pyrrolidine alkaloids were investigated for their antimycobacterial activities against* M. tuberculosis*, where many of them exhibited a notable and potent efficacy and may be regarded as lead molecules for the treatment of tuberculosis [48].

In the present study, we investigated the acute and chronic toxicity of both plant species in animal model. The treatment of animals with doses lower than 5 g/kg of all extracts in acute toxicity test did not show any mortality or observable symptoms. The mortality rate and LD_50_ values were calculated. Surprisingly,* A. galanga* showed more toxicity than* T. cordifolia*. In the chronic toxicity test, we evaluated the extracts effects on body weight, food and water consumption, organ weight, haematological and biochemical parameters, and lipid profile. After 12 weeks of treatment, it was observed that both plants have no effect on the body weight or food and water consumption but* A. galanga* caused significantly an increase in the relative weight of the heart, the liver, the spleen, and kidney as compared to the control group. The rise in organ weight could be referred to stimulation of xenobiotic enzymes promoting to raise in proteins synthesis. Regularly the inducement of these enzymes leads to a raise of relative organ weight following an exposure to xenobiotic [49, 50]. It could be argued that these alterations might be toxicological significant particularly for heart, liver, and kidney but the raise in the relative weight of spleen might be due to the high unevenness of the weight of this organ [51]. Haematological studies of both plants revealed a significant fall in the RBC and WBC level as well as haemoglobin and platelets. Our results were not in agreement with data reported previously by Qureshi and coworkers [52] who demonstrated a significant rise in the RBC level of* A. galanga*-treated animals. In addition,* A. galanga* extracts increased significantly all liver enzymes (AST, ALT, GGT, and ALU) which could indicate a liver damage or injury. The significant increase of blood glucose level by* A. galanga* extracts could be attributed to insufficiency in the storage of glucose or to demolition of *β*–cells of the pancreas. In our study, the prolonged administration of the extracts showed a large variation in the lipid profile which could be attributed to the nature of the constituents in those extracts and might be connected to liver damage.

## 5. Conclusion

The results reported in this study are quite interesting showing considerable antimycobacterial activity of* A. galanga* and* T. cordifolia*. The antimycobacterial activity is consistent with traditional use in the treatment of cough-diseases including tuberculosis. Moreover, our results have exhibited that* A. galanga* and* T. cordifolia* might be toxic to heart, liver, and kidney after long-term administration. Thus, some caution should be taken into consideration when these plant species are administered for long periods.

## Figures and Tables

**Table 1:** Results of extraction of the plants and phytochemical screening.

Plants	Part used	Solvent	Yield in % (g)	Phytochemical screening
*Alpinia galanga*	roots & rhizomes	Dichloromethane	4.2 (115)	terpenoids, essential oil,
	roots & rhizomes	Ethanol	1.0 (26)	terpenoids, flavonoids, phenolics
*Tinospora cordifola*	leaves & stems	Dichloromethane	2.2 (54)	terpenoids, flavonoids
	leaves & stems	Ethanol	1.5 (35)	flavonoids, alkaloids

**Table 2:** Mortality rate and LD-50 values of the investigated *Alpinia galanga* and *Tinospora cordifolia* extracts in mice.

**Treatments** **(n=6)**	**Mortality rate of animals (at different plant extract doses in g/kg, i.p.)**						**LD**_**50**_ ** by Karber's method**
	**0.5**	**1**	**2**	**5**	**8**	**10**	
Control	No death	No death	No death	No death	No death	No death	
AGDC	No death	No death	No death	1	4	6	6.66
AGET	No death	No death	No death	No death	2	4	7.75
TCDC	No death	No death	No death	No death	2	4	7.58
TCET	No death	No death	No death	2	3	4	6.83

AGDC:* Alpinia galanga* dichloromethane extract; AGET: *Alpinia galanga* ethanol extract; TCDC: *Tinospora cordifolia* dichloromethane extract; TCET: *Tinospora cordifolia* ethanol extract.

**Table 3:** Effect of the extracts on the relative organ weight of mice after 12 weeks treatment.

Parameters	Extracts								
	Control	AGDC	%	AGET	%	TCDC	%	TCET	%
		166 mg/kg	change	193 mg/kg	change	189 mg/kg	change	170 mg/kg	change
Heart	0.11 ± 0.006	0.15 ± 0.004*∗∗*	36↑	0.15 ± 0.01*∗∗*	36↑	0.13 ± 0.006	18↑	0.12 ± 0.008	9↑
Liver	1.29 ± 0.07	1.47 ± 0.02*∗∗*	14↑	1.48 ± 0.01*∗∗∗*	15↑	1.14 ± 0.32	11↓	1.46 ± 0.02*∗∗*	13↑
Lungs	0.39 ± 0.01	0.45 ± 0.001*∗*	15↑	0.44 ± 0.02	12↑	0.36 ± 0.006	8↓	0.40 ± 0.01	2↑
Spleen	0.11 ± 0.007	0.15 ± 0.01*∗*	36↑	0.12 ± 0.01*∗*	9↑	0.13 ± 0.006	18↑	0.13 ± 0.007	18↑
Kidney	0.32 ± 0.01	0.38 ± 0.01*∗*	18↑	0.44 ± 0.01*∗∗∗*	37↑	0.33 ± 0.003	3↑	0.44 ± 0.008*∗∗∗*	37↑
Testis	0.23 ± 0.007	0.26 ± 0.01	13↑	0.28 ± 0.01*∗*	21↑	0.23 ± 0.02		0.27 ± 0.02	17↑

All values represent mean ± SEM. *∗p *< 0.05; *∗∗p* < 0.01; *∗∗∗p* < 0.001; ANOVA, followed by Dunnett's multiple comparison test. Test value compared with control group

**Table 4:** Effect of the extracts on haematological parameters of mice in chronic toxicity test.

Parameters	Extracts								
	Control	AGDC	%	AGET	%	TCDC	%	TCET	%
		166 mg/kg	change	193 mg/kg	change	189 mg/kg	change	170 mg/kg	change
RBC (x 10^6^/mm^3^)	7.8 ± 0.12	6.80 ± 0.12*∗∗*	13↓	6.35 ± 0.15*∗∗∗*	18↓	7.00 ± 0.20	10↓	6.92 ± 0.08*∗∗*	11↓
WBC (x 10^3^/mm^3^)	9.10 ± 0.17	8.45 ± 0.06*∗*	7↓	6.97 ± 0.11*∗∗*	23↓	9.17 ± 0.12	-	7.77 ± 0.19*∗∗*	15↓
Hemoglobin (g/dl)	11.75 ± 0.19	11.0 ± 0.20	6↓	9.05 ± 0.19*∗∗∗*	23↓	11.45 ± 0.42	2↓	10.32 ± 0.19*∗∗*	12↓
Platelets( x 10^3^/mL)	276.7 ± 4.32	263.0 ± 3.24	5↓	244.7 ± 3.37*∗∗*	12↓	255.0 ± 2.58*∗*	8↓	260.25 ± 2.68*∗*	6↓
Neutrophils (x 10^3^/mm^3^)	3.70 ± 0.09	3.15 ± 0.14	15↓	3.35 ± 0.06	9↓	3.45 ± 0.15	7↓	3.30 ± 0.25	11↓
Lymphocytes ( x 10^3^/mm^3^)	5.95 ± 0.11	5.52 ± 0.23	7↓	5.67 ± 0.18	5↓	5.37 ± 0.25	10↓	4.87 ± 0.12*∗∗∗*	18↓

All values represent mean ± SEM. *∗p *< 0.05; *∗∗p* < 0.01; *∗∗∗p* < 0.001; ANOVA, followed by Dunnett's multiple comparison test. Test value compared with control group

**Table 5:** Effect of the extracts on biochemical parameters of mice in chronic toxicity test.

Parameters	Extracts								
	Control	AGDC	%	AGET	%	TCDC	%	TCET	%
		166 mg/kg	change	193 mg/kg	change	189 mg/kg	change	170 mg/kg	change
AST(U/l)	102.75 ± 5.21	128.25 ± 6.10*∗*	26↑	134.0 ± 4.14*∗∗*	31↑	98.45 ± 5.18	4↓	131.0 ± 5.36*∗∗*	28↑
ALT(U/l)	30.62 ± 2.14	47.97 ± 1.42*∗∗∗*	56↑	53.92 ± 4.45*∗∗*	76↑	26.7 ± 1.64	13↓	39.62 ± 2.93	35↑
GGT(U/l)	4.75 ± 0.15	6.72 ± 0.23*∗∗∗*	42↑	5.75 ± 0.29*∗*	21↑	3.12 ± 0.08*∗∗∗*	34↓	4.6 ± 0.28	3↓
ALP(U/l)	320.75 ± 9.64	414.0 ± 8.39*∗∗∗*	29↑	425.0 ± 6.64*∗∗∗*	32↑	347.50 ± 7.55	8↑	385.5 ± 6.06*∗*	12↑
Bilirubin(mg/dl)	0.52 ± 0.01	0.77 ± 0.01*∗∗∗*	47↑	0.71 ± 0.02*∗∗*	36↑	0.50 ± 0.01		0.68 ± 0.02*∗∗*	30↑
Glucose(mg/dl)	122.0 ± 4.88	196.25 ± 7.18*∗∗∗*	61↑	136.25 ± 6.26	11↑	77.90 ± 0.02*∗∗*	36↓	123.50 ± 8.45	
Sodium (mEq/L)	84.18 ± 1.97	107.14 ± 2.8*∗∗∗*	27↑	99.10 ± 2.36*∗∗*	17↑	117.47 ± 3.9*∗∗*	40↑	105.99 ± 1.9*∗∗∗*	26↑
Potassium (mEq/L)	5.0 ± 0.02	7.22 ± 0.28*∗∗∗*	45↑	6.97 ± 0.11*∗∗∗*	40↑	6.6 ± 0.12*∗∗∗*	32↑	5.60 ± 0.12	12↑
Calcium (mg/dl)	7.42 ± 0.59	5.61 ± 0.66	24↓	5.52 ± 0.65	26	5.23 ± 0.42*∗*	29↓	6.95 ± 0.47	6↓
Urea (nmol/l)	39.1 ± 1.78	38.95 ± 1.41		29.92 ± 3.50	23↓	59.35 ± 4.26*∗∗*	52↑	65.0 ± 3.53*∗∗∗*	66↑
Uric acid (mg/dl)	6.97 ± 0.25	4.95 ± 0.09*∗∗∗*	29↓	8.87 ± 0.30*∗∗*	27↑	6.87 ± 0.30		10.27 ± 0.45*∗∗∗*	47↑
Creatinine (mg/dl)	0.92 ± 0.04	1.01 ± 0.04	9↑	1.27 ± 0.02*∗∗∗*	38↑	1.44 ± 0.02*∗∗∗*	56↑	1.52 ± 0.09*∗∗∗*	65↑

All values represent mean ± SEM. *∗p *< 0.05; *∗∗p* < 0.01; *∗∗∗p* < 0.001; ANOVA, followed by Dunnett's multiple comparison test. Test value compared with control group

**Table 6:** Effect of the extracts on lipid profile and total protein of male and female mice in chronic toxicity test.

Parameters	Extracts								
	Control	AGDC	%	AGET	%	TCDC	%	TCET	%
		166 mg/kg	change	193 mg/kg	change	189 mg/kg	change	170 mg/kg	change
Cholesterol	108.0 ± 3.43	170.75 ± 4.4*∗∗∗*	58↑	85.7 ± 4.37*∗*	21↓	77.9 ± 4.95*∗∗*	28↓	133.75 ± 3.42*∗∗*	24↑
Triglycerides(mg/dl)	60.6 ± 2.29	147.0 ± 5.36*∗∗∗*	143↑	39.92 ± 1.38*∗∗∗*	34↓	43.71 ± 2.24*∗∗*	28↓	119.25 ± 6.1*∗∗∗*	97↑
HDL(mg/dl)	51.0 ± 0.87	87.55 ± 5.4*∗∗∗*	72↑	37.82 ± 2.21*∗∗*	26↓	39.25 ± 1.2*∗∗∗*	23↓	82.1 ± 3.3*∗∗∗*	61↑
VLDL(mg/dl)	12.12 ± 0.45	29.4 ± 107*∗∗∗*	143↑	7.98 ± 0.27*∗∗∗*	34↓	8.75 ± 0.44*∗∗*	28↓	23.85 ± 1.23*∗∗∗*	97↑
LDL(mg/dl)	44.64 ± 4.35	60.3 ± 6.66	35↑	35.34 ± 1.53	20↓	26.47 ± 1.33*∗∗*	41↓	28.67 ± 4.98*∗*	36↓
Total Protein (g/dl)	5.3 ± 0.14	3.05 ± 0.11*∗∗∗*	42↓	3.7 ± 0.18*∗∗∗*	30↓	6.25 ± 0.40	18↑	3.67 ± 0.320*∗∗∗*	31↓

All values represent mean ± SEM. *∗p *< 0.05; *∗∗p* < 0.01; *∗∗∗p* < 0.001; ANOVA, followed by Dunnett's multiple comparison test. Test value compared with control group

## Data Availability

The data used to support the findings of this study are available from the corresponding author upon request.

## References

[1] Jordao L., Vieira O. V. (2011). Tuberculosis: new aspects of an old disease. *International Journal of Cell Biology*.

[2] Sabran S. F., Mohamed M., Abu Bakar M. F. (2016). Ethnomedical knowledge of plants used for the treatment of tuberculosis in Johor, Malaysia. *Evidence-Based Complementary and Alternative Medicine*.

[3] Organization W. H. (2016). Global tuberculosis report 2016.

[4] Nguta J. M., Appiah-Opong R., Nyarko A. K., Yeboah-Manu D., Addo P. G. A. (2015). Medicinal plants used to treat TB in Ghana. *International Journal of Mycobacteriology*.

[5] Kahaliw W., Aseffa A., Abebe M., Teferi M., Engidawork E. (2017). Evaluation of the antimycobacterial activity of crude extracts and solvent fractions of selected Ethiopian medicinal plants. *BMC Complementary and Alternative Medicine*.

[6] Mitscher L. A., Baker W. R. (1998). A search for novel chemotherapy against tuberculosis amongst natural products. *Pure and Applied Chemistry*.

[7] Juntachote T., Berghofer E., Siebenhandl S., Bauer F. (2006). The antioxidative properties of Holy basil and Galangal in cooked ground pork. *Meat Science*.

[8] Gupta P., Bhatter P., D'souza D. (2014). Evaluating the anti Mycobacterium tuberculosis activity of Alpinia galanga (L.) Willd. axenically under reducing oxygen conditions and in intracellular assays. *BMC Complementary and Alternative Medicine*.

[9] Elyani H., Risandiansyah R. (2017). Antibacterial potential of four herbal plants (Syzygium cumini, Piper ornatum, Anredera cordifolia, and Alpinia galanga) against Staphylococcus aureus and Escherichia coli. *JIMR-Journal of Islamic Medicine Research*.

[10] Sanusi S. B., Bakar A., Fadzelly M., Mohamed M., Sabran S. F., Mainasara M. M. (2017). Southeast asian medicinal plants as a potential source of antituberculosis agent. *Evidence-Based Complementary and Alternative Medicine*.

[11] Verma R., Mishra G., Singh P., Jha K., Khosa R. (2011). *Alpinia galanga*–An important medicinal plant: a review. *Der Pharmacia Sinica*.

[12] Shetty G. R., Monisha S. (2015). Pharmacology of an endangered medicinal plant *Alpinia galanga*-a review. *Research Journal of Pharmaceutical, Biological and Chemical Sciences*.

[13] Choudhary N., Siddiqui M., Azmat S., Khatoon S. (2013). *Tinospora cordifolia*: ethnobotany, phytopharmacology and phytochemistry aspects. *International Journal of Pharmaceutical Sciences and Research*.

[14] Badar V. A., Thawani V. R., Wakode P. T. (2005). Efficacy of *Tinospora cordifolia* in allergic rhinitis. *Journal of Ethnopharmacology*.

[15] Krishna K., Jigar B., Jagruti P. (2009). Guduchi (*Tinospora cordifolia*): Biological and medicinal properties, a review. *The Internet Journal of Alternative Medicine*.

[16] Kaushik A., Husain A., Awasthi H., Singh D. P., Khan R., Mani D. (2017). Antioxidant and hepatoprotective potential of Swaras and Hima extracts of Tinospora cordifolia and Boerhavia diffusa in Swiss albino mice. *Pharmacognosy Magazine*.

[17] Polu P. R., Nayanbhirama U., Khan S., Maheswari R. (2017). Assessment of free radical scavenging and anti-proliferative activities of Tinospora cordifolia Miers (Willd). *BMC Complementary and Alternative Medicine*.

[18] Bansal P., Malik M., Das S., Kaur J. (2017). Tinospora cordifolia induces cell cycle arrest in human oral squamous cell carcinoma cells. *The Gulf Journal of Oncology*.

[19] Sharma N., Kumar A., Sharma P. R. (2018). A new clerodane furano diterpene glycoside from Tinospora cordifolia triggers autophagy and apoptosis in HCT-116 colon cancer cells. *Journal of Ethnopharmacology*.

[20] Haque M. A., Jantan I., Abbas Bukhari S. N. (2017). Tinospora species: An overview of their modulating effects on the immune system. *Journal of Ethnopharmacology*.

[21] Dhama K., Sachan S., Khandia R. (2017). Medicinal and beneficial health applications of tinospora cordifolia (Guduchi): A miraculous herb countering various diseases/disorders and its immunomodulatory effects. *Recent Patents on Endocrine, Metabolic & Immune Drug Discovery*.

[22] Wagner H., Bladt S. (1996). *Plant Drug Analysis: A Thin Layer Chromatography Atlas*.

[23] OECD (1994). *OECD Guidelines for the Testing of Chemicals*.

[24] Shah A. H., Qureshi S., Tariq M., Ageel A. M. (1989). Toxicity studies on six plants used in the traditional Arab system of medicine. *Phytotherapy Research*.

[25] Walker B. R., Colledge N. R. (2013). *Davidson's Principles and Practice of Medicine E-Book*.

[26] Daniel W. W., Cross C. L. (1995). *Biostatistics: A Foundation for Analysis in The Health Sciences*.

[27] Organization W. H. (2013). *Global tuberculosis report 2013*.

[28] Nguta J. M., Appiah-Opong R., Nyarko A. K., Yeboah-Manu D., Addo P. G. A. (2015). Current perspectives in drug discovery against tuberculosis from natural products. *International Journal of Mycobacteriology*.

[29] Narayanan A. S., Raja S. S. S., Ponmurugan K. (2011). Antibacterial activity of selected medicinal plants against multiple antibiotic resistant uropathogens: A study from Kolli Hills, Tamil Nadu, India. *Beneficial Microbes*.

[30] Nipanikar S., Chitlange S., Nagore D. (2017). Evaluation of anti-inflammatory and antimicrobial activity of AHPL/AYCAP/0413 capsule. *Pharmacognosy Research*.

[31] Soundhari C., Rajarajan S. (2013). In vitro screening of lyophilised extracts of Alpinia galanga L. and Oldenlandia umbellata L. for antimycobacterial activity. *International Journal of Biological and Pharmaceutical Research*.

[32] Warit S., Rukseree K., Prammananan T. (2017). In vitro activities of enantiopure and racemic 1′-acetoxychavicol acetate against clinical isolates of mycobacterium tuberculosis. *Scientia Pharmaceutica*.

[33] Gupta P. K., Chakraborty P., Kumar S. (2016). G1-4A, a polysaccharide from *Tinospora cordifolia* inhibits the survival of *Mycobacterium tuberculosis* by modulating host immune responses in TLR4 dependent manner. *PLoS ONE*.

[34] Manse Y., Ninomiya K., Nishi R. (2017). Labdane-type diterpenes, galangalditerpenes A–C, with melanogenesis inhibitory activity from the fruit of alpinia galanga. *Molecules*.

[35] Kaur A., Singh R., Dey C. S., Sharma S. S., Bhutani K. K., Singh I. P. (2010). Antileishmanial phenylpropanoids from Alpinia galanga (Linn.) Willd. *Indian Journal of Experimental Biology*.

[36] Abdullah F., Subramanian P., Ibrahim H., Malek S. N. A., Lee G. S., Hong S. L. (2015). Chemical composition, antifeedant, repellent, and toxicity activities of the rhizomes of galangal, alpinia galanga against asian subterranean termites, coptotermes gestroi and coptotermes curvignathus (Isoptera: Rhinotermitidae). *Journal of Insect Science*.

[37] Tadtong S., Watthanachaiyingcharoen R., Kamkaen N. (2014). Antimicrobial constituents and synergism effect of the essential oils from Cymbopogon citratus and Alpinia galanga. *Natural Product Communications (NPC)*.

[38] Patel M. B., Mishra S. (2012). Isoquinoline alkaloids from tinospora cordifolia inhibit rat lens aldose reductase. *Phytotherapy Research*.

[39] Van Kiem P., Van Minh C., Dat N. T. (2010). Aporphine alkaloids, clerodane diterpenes, and other constituents from *Tinospora cordifolia*. *Fitoterapia*.

[40] Nyandoro S. S., Munissi J. J. E., Kombo M. (2017). Flavonoids from Erythrina schliebenii. *Journal of Natural Products*.

[41] Jnawali H. N., Jeon D., Jeong M.-C. (2016). Antituberculosis Activity of a Naturally Occurring Flavonoid, Isorhamnetin. *Journal of Natural Products*.

[42] Christopher R., Nyandoro S. S., Chacha M., De Koning C. B. (2014). A new cinnamoylglycoflavonoid, antimycobacterial and antioxidant constituents from Heritiera littoralis leaf extracts. *Natural Product Research (Formerly Natural Product Letters)*.

[43] McCulloch M. W. B., Haltli B., Marchbank D. H., Kerr R. G. (2012). Evaluation of pseudopteroxazole and pseudopterosin derivatives against Mycobacterium tuberculosis and other pathogens. *Marine Drugs*.

[44] Isaka M., Chinthanom P., Sappan M. (2017). Antitubercular activity of mycelium-associated ganoderma lanostanoids. *Journal of Natural Products*.

[45] Isaka M., Chinthanom P., Sappan M., Danwisetkanjana K., Boonpratuang T., Choeyklin R. (2016). Antitubercular Lanostane Triterpenes from Cultures of the Basidiomycete Ganoderma sp. BCC 16642. *Journal of Natural Products*.

[46] Ramos Alvarenga R. F., Wan B., Inui T., Franzblau S. G., Pauli G. F., Jaki B. U. (2014). Airborne antituberculosis activity of Eucalyptus citriodora essential oil. *Journal of Natural Products*.

[47] Gu J.-Q., Wang Y., Franzblau S. G., Montenegro G., Timmermann B. N. (2004). Constituents of Senecio chionophilus with potential antitubercular activity. *Journal of Natural Products*.

[48] Mishra S. K., Tripathi G., Kishore N., Singh R. K., Singh A., Tiwari V. K. (2017). Drug development against tuberculosis: Impact of alkaloids. *European Journal of Medicinal Chemistry*.

[49] Diallo A., Eklu-Gadegbeku K., Amegbor K. (2014). In vivo and in vitro toxicological evaluation of the hydroalcoholic leaf extract of Ageratum conyzoides L. (Asteraceae). *Journal of Ethnopharmacology*.

[50] Irene I. I., Chukwunonso C. A. (2006). Body and organ weight changes following administration of aqueous extracts of Ficus exasperata. Vahl on white albino rats. *Journal of Animal and Veterinary Advances*.

[51] Popp J. A. (2005). *Best Practice for The Routine Pathology Evaluation of The Immune System*.

[52] Qureshi S., Shah A. H., Ageel A. M. (1992). Toxicity studies on Alpinia galanga and Curcuma longa. *Planta Medica*.

